# Draft Genome Sequence of *Lactobacillus mulieris* UMB9245, Isolated from the Female Bladder

**DOI:** 10.1128/MRA.00408-20

**Published:** 2020-05-14

**Authors:** Lidia Ramotowski, Taylor Miller-Ensminger, Adelina Voukadinova, Alan J. Wolfe, Catherine Putonti

**Affiliations:** a Department of Biology, Loyola University Chicago, Chicago, Illinois, USA; b Bioinformatics Program, Loyola University Chicago, Chicago, Illinois, USA; c Department of Microbiology and Immunology, Stritch School of Medicine, Loyola University Chicago, Maywood, Illinois, USA; d Department of Computer Science, Loyola University Chicago, Chicago, Illinois, USA; DOE Joint Genome Institute

## Abstract

Lactobacillus jensenii is an anaerobic bacterium found in the urogenital tract that is known to prevent common vaginal infections. Recently, it was divided into two species, L. jensenii and L. mulieris. Here, we report the draft genome sequence of L. mulieris UMB9245, with a genome length of 1,723,383 bp assembled into 52 contigs.

## ANNOUNCEMENT

Species of *Lactobacillus*, particularly L. crispatus, L. gasseri, L. iners, and L. jensenii, are common members of the “healthy” female urogenital microbiota (1, 2). Evidence suggests that L. jensenii can prevent common infections, e.g., urinary tract infections (UTIs) and sexually transmitted infections, among others (3–5). L. jensenii acts as a barrier against pathogens and protects against various urogenital infections by outcompeting foreign invaders (6, 7). Among the lactobacilli, L. jensenii is one of the species most commonly isolated from the urogenital tract (8, 9). Recently, the original species designation of L. jensenii was split into two species with the designation of a new species, L. mulieris (10). The L. mulieris type species was isolated from a urine sample; here, we present the draft genome sequence of another urinary L. mulieris strain. L. mulieris UMB9245 was isolated from a catheterized urine sample obtained from a female patient with a UTI.

L. mulieris UMB9245 was isolated from a prior institutional review board (IRB)-approved study (University of California, San Diego, IRB no. 170077AW) using the expanded quantitative urinary culture (EQUC) protocol (11). The genus and species were confirmed by matrix-assisted laser desorption ionization–time of flight (MALDI-TOF) mass spectrometry, as described previously (11). The isolates were then stored at −80°C until sequencing. The isolate was streaked onto a Columbia naladixic acid (CNA) agar plate and incubated for 24 h at 35°C in 5% CO_2_. A single colony was selected and grown in a liquid culture using MRS medium supplemented with 1 ml/liter Tween 80 under the same conditions as described above. DNA extraction was done using the Qiagen DNeasy blood and tissue kit following the manufacturer’s protocol for Gram-positive bacteria, with the following alterations: we used 230 μl of lysis buffer (180 μl of 20 mM Tris-Cl, 2 mM sodium EDTA, and 1.2% Triton X-100 and 50 μl of lysozyme) in step 2 and altered the incubation time in step 5 to 10 min. The DNA was quantified using a Qubit fluorometer and sent to the Microbial Genomic Sequencing Center at the University of Pittsburgh for library preparation (Illumina Nextera chemistry) and sequencing on the Illumina NextSeq 550 platform, which produced 2,972,316 pairs of 2 × 150-bp reads. Raw reads were trimmed using Sickle v1.33 (https://github.com/najoshi/sickle) and assembled using SPAdes v3.13.0 with the “only-assembler” option for k values of 55, 77, 99, and 127 (12). The genome coverage was calculated using BBMap v38.47 (https://sourceforge.net/projects/bbmap). We initially annotated our genome assembly using PATRIC v3.6.3 (13). The publicly available genome was annotated using the NCBI Prokaryotic Genome Annotation Pipeline (PGAP) (14).

The L. mulieris UMB9245 draft genome is 1,723,383 bp long, assembled into 52 contigs with a coverage of 405×, GC content of 34.18%, and *N*_50_ value of 66,175 bp. Our annotations identified 6 rRNAs (3 5S rRNAs, 2 16S rRNAs, and 1 23S rRNA) and 53 tRNAs. Furthermore, three intact prophages were identified by PHASTER (15), two resembling Lactobacillus jensenii-infecting phage Lv-1 (GenBank accession no. NC_011801) and one resembling Lactobacillus johnsonii-infecting phage Lj928 (GenBank accession no. NC_005354). Each of the predicted intact phage sequences was then queried against the nonredundant/nucleotide database via BLASTn, confirming the PHASTER-predicted homology to Lv-1 (70% query coverage and 93.21% sequence identity for one phage and 3% query coverage and 74.97% sequence identity for another phage) and Lj928 (15% query coverage and 69.02% sequence identity). One CRISPR array, containing 42 spacer sequences, was detected by CRISPRFinder (16).

Future analyses of the newly defined species L. mulieris will further our understanding of this bacterium, particularly with respect to its potential role in the urinary microbiome.

### Data availability.

This whole-genome shotgun project has been deposited in GenBank under accession no. JAAVSE000000000. The version described in this paper is the first version, JAAVSE010000000. The raw sequencing reads have been deposited in the SRA under accession no. SRR11441021.
